# Comparison of wavelength selection methods for in-vitro estimation of lactate: a new unconstrained, genetic algorithm-based wavelength selection

**DOI:** 10.1038/s41598-020-73406-4

**Published:** 2020-10-09

**Authors:** Mohammad Mamouei, Karthik Budidha, Nystha Baishya, Meha Qassem, Panayiotis Kyriacou

**Affiliations:** Research Centre for Biomedical Engineering, Department of Electrical and Electronic Engineering, School of Mathematics, Computer Science and Engineering, City, University of London, London, UK

**Keywords:** Applied mathematics, Computational science, Near-infrared spectroscopy, Predictive markers

## Abstract

Biochemical and medical literature establish lactate as a fundamental biomarker that can shed light on the energy consumption dynamics of the body at cellular and physiological levels. It is therefore, not surprising that it has been linked to many critical conditions ranging from the morbidity and mortality of critically ill patients to the diagnosis and prognosis of acute ischemic stroke, septic shock, lung injuries, insulin resistance in diabetic patients, and cancer. Currently, the gold standard for the measurement of lactate requires blood sampling. The invasive and costly nature of this procedure severely limits its application outside intensive care units. Optical sensors can provide a non-invasive, inexpensive, easy-to-use, continuous alternative to blood sampling. Previous efforts to achieve this have shown significant potential, but have been inconclusive. A measure that has been previously overlooked in this context, is the use of variable selection methods to identify regions of the optical spectrum that are most sensitive to and representative of the concentration of lactate. In this study, several wavelength selection methods are investigated and a new genetic algorithm-based wavelength selection method is proposed. This study shows that the development of more accurate and parsimonious models for optical estimation of lactate is possible. Unlike many existing methods, the proposed method does not impose additional locality constraints on the spectral features and therefore helps provide a much more granular interpretation of wavelength importance.

## Introduction

Lactate is a valuable fundamental biomarker that can inform the diagnosis and prognosis of many critical conditions. It has been linked to morbidity and mortality of critically ill patients^1,2^, acute ischemic stroke ^3–5^, septic shock^6,7^, lung injuries^8^, insulin resistance in diabetic patients^9^, and cancer ^9–12^. Moreover, it has been suggested that monitoring lactate levels can shed light on the performance of important organs such as the brain^13^ and the heart^14^. Finally, the lactate shuttle theory highlights the significance of this biomarker in understanding the energy consumption dynamics of the body as a whole^15^.

The clinically accepted procedure for the measurement of blood lactate relies on intermittent blood sampling. However, the invasive and costly nature of this procedure severely limits its application outside intensive care units. The development of sensors that build on the optical properties of lactate to estimate its concentration, can lead to a non-invasive, transcutaneous, continuous, and inexpensive alternative to blood sampling. Some devices are commercially available for this purpose, but they produce indirect and inaccurate measurements of lactate by correlating it with muscle oxygen saturation, SmO_2_. There have been a limited number of studies targeting direct, optical estimation of lactate. These studies, in spite of underlining the potential for optical measurements of lactate, have not been conclusive.

A study on quantification of lactate in plasma and using Fourier Transform InfraRed (FT-IR) spectroscopy reported promising results^16^. Therein, Based on an iterative procedure, the concentration of an analyte is estimated from the sample’s absorption profile. Subsequently, the analyte’s contribution to the absorption profile is subtracted and the next analyte is estimated. Using a validation set of 14 plasma samples from 14 different patients, a coefficient of correlation of ${r}_{v}$ =0.97 and a Root-Mean-Square Error (RMSE) of Validation (RMSEV) of 0.15 mmol/l was reported for the estimation of lactate. Similarly, the use of multivariate models, namely Partial Least Squares (PLS), has been investigated to estimate the concentration of lactate in Near InfraRed (NIR) spectra of whole human blood^17^. Therein, the spectra covered the wavelength range of 2050–2400 nm and were collected in transmittance mode. Three blood samples were collected from each of the five participating subjects during an exercise study. Each sample was spiked twice with lactate to produce a wider range of lactate variations in the dataset; yielding 45 samples with different lactate concentrations. Using leave-one-out cross-validation, a correlation coefficient of ${r}_{CV}=0.978$ and a RMSE of Cross-Validation (RMSECV) of 0.65 mmol/l were reported between the predicted values and the reference values. A baseline correction by subtracting each subject’s baseline spectrum from its spectra was shown to significantly improve the results; leading to ${r}_{CV}$ = 0.992 and RMSECV of 0.21 mmol/l. Following this, the in-vivo application of this method was carried out within the 1500–1750 nm spectral range^18^. Forty samples were collected from 10 subjects in an excericse study and their spectra were used for training and cross-validation. A correlation coefficient of ${r}_{CV}=0.98$ and a RMSECV = 0.76 mmol/l were reported. Without the inter-subject baseline correction, these values were ${r}_{cv}\hspace{0.17em}=\hspace{0.17em}$0.86 and RMSECV = 2.21 mmol/l. This clearly highlights the importance of baseline differences between subjects, both in blood composition and in the optical properties of the skin.

While the aforementioned studies clearly demonstrate the potential of multivariate modelling and optical spectroscopy in achieving a reagent-free and potentially non-invasive measurement of lactate, to date, a direct and accurate optical lactate sensor has not yet been developed. The authors believe that a comprehensive analysis of the optical properties of lactate and the identification of wavelengths that are most indicative of its concentration can play a crucial role in achieving this aim. A data-driven approach to fulfil this objective is the use of variable (wavelength) selection methods. These methods offer two clear benefits. Firstly, it has been shown that the inclusion of uninformative wavelengths in the training process negatively affects the accuracy of predictions and model interpretability^19–22^. Secondly, from a more practical point of view, the identification of a few wavelengths, or regions of the optical spectrum, that contain information about chemical species, significantly reduces the time and cost associated with their measurement and enables the development of portable and high-speed optical sensors. In spite of these benefits, no study in the literature has investigated the application of wavelength selection methods for the estimation of lactate. To this end, this study conducts a comprehensive analysis of the application of wavelength selection methods on a set of spectra that were obtained by controlled variation of Na-lactate in a Phosphate Buffer Solution (PBS). PBS was chosen to firstly ensure minimal chemical changes that may be correlated with the concentration of lactate, and secondly to facilitate the interpretation of results.

The rest of this article is organised as follows. Section 2, highlights the importance of lactate as a fundamental biomarker. This section is, then, concluded with a brief on spectroscopic data and quantitative methods for their analysis. Section 3 describes the lactate and PBS dataset. Section 4 provides a summary of three classes of wavelength selection methods, describes the four methods investigated in this study, outlines their limitations, and presents the new GA algorithm. Finally, Sect. 5 reports the results and concludes this paper.

## Background

### Cellular respiration, lactate, and adenosine triphosphate

Highlighting the relationship between lactate and Adenosine TriPhosphate (ATP), known as the universal energy currency of cells, helps demonstrate the importance of lactate as a remarkable biomarker. Transcending human biology, ATP is seen in all living organisms. It provides cells with easily accessible energy to fuel various processes such as biosynthesis, metabolism, DeoxyriboNucleic Acid (DNA) synthesis, muscle contraction, transport of ions and impulse transition in the nervous system^23^. The storage of easily accessible bioenergy in ATP is the outcome of cellular respiration. In this process, carbohydrates are broken down into simple sugars and these simple sugars are further processed to produce ATP. In animals, including humans, glucose is the most common type of such sugars that can be metabolised by cells.

Glycolysis is an important ATP producing system in living organisms. In glycolysis, cells break down a molecule of glucose and extract a net of two ATP and two molecules of pyruvate. This process does not require oxygen (anaerobic) and can produce ATP at a very fast rate. For instance, in an intense maximal workout, glycolysis may become an important energy production mechanism to fuel the body, but only for a few minutes. In the absence of a sufficient supply of oxygen, pyruvate is converted to lactate to help maintain glycolysis for longer periods. In the presence of oxygen, pyruvate, together with fatty acids and amino acids, are eventually reduced to hydrogen and carbon dioxide in a chain of reactions that produce around 28–30 additional ATP per molecule of glucose. The whole system is collectively referred to as aerobic respiration and includes 4 stages; glycolysis, the link reaction, the Krebs cycle, and oxidative phosphorylation^24^.

The completion of an aerobic respiration cycle, takes a longer time than glycolysis alone, up to 100 times. It is, however, much more “efficient” in terms of ATP production, as 30–32 ATPs per molecule of glucose are produced in the aerobic cycle compared to 2 ATPs per molecule of glucose in glycolysis alone^25^. But of course, among other things, aerobic respiration requires sufficient supplies of oxygen. Therefore, higher than normal levels of glycolysis, and consequently, higher levels of lactate may be observed in conditions where the oxygen supply to cells is restricted or when cells need faster than normal deposits of energy relative to what aerobic respiration can supply.

This brief introduction highlights the importance of lactate as a fundamental biomarker that sheds light on the energy consumption patterns of the body. What follows provides a brief overview of the role of lactate as a prognostic and diagnostic measure in a wide range of diseases.

### Lactate: a valuable indicator of disease

Lactate is a valuable biomarker in understanding diseases both at the cellular and physiological levels. From a cellular perspective, as described in Sect. 2.1, lactate can be indicative of the energy consumption dynamics. From a physiological perspective, some organs are -mainly- lactate consuming; for instance, it has been suggested that lactate may be an efficient fuel for the brain. Some organs are lactate clearing; for instance, the liver processes excess, non-metabolised lactate and converts it back to glucose in the Cori Cycle. Finally, some organs are lactate producing such as skeletal muscles and some are lactate clearing such as the kidney. The kidney helps clear excess lactate in hyperlactatemia. The lactate shuttle theory describes a dynamic, pH-dependant flow of lactate between organs^15^. Therefore, a major, unexpected disruption in these dynamics can be an early sign of disease.

While such dynamics are not fully understood, the relationship between lactate and many diseases is well-documented. Insufficient delivery of oxygen to tissue (hypoxia) or insufficient perfusion of the tissue (ischemia) can have drastic consequences. The most significant tissues that are commonly affected by hypoxia or ischemia are cardiac and cerebral tissues. Severe cardiac or cerebral ischemia can cause irreversible and potentially fatal conditions, namely myocardial infarction and acute ischemic stroke. As mentioned in Sect. 1.1, insufficient oxygen delivery can also tilt the healthy balance between glycolysis and oxidative pathways towards the former, and result in high levels of lactate (hyperlactatemia). Therefore, monitoring lactate levels can contribute to improved diagnosis and prognosis in these conditions^3,5,26^. Similarly, different types of shock, caused by inadequate cardiac pump function (cardiogenic shock), severe infection (septic shock), obstruction of the vessels (obstructive shock), and decrease in blood volume (hypovolemic shock) are associated with hyperlactatemia^6,14^.

Increased lactate levels have been observed in patients with lung injury^8^, increased white blood cell activity^27^, and reduced lactate clearance capability by the liver and kidney^28^. Lactate has been shown to be an important diagnostic marker of generalised tonic–clonic seizures^29^. Increased lactate level has been linked to the progression of insulin resistance in diabetic patients^9^. Lactate has also been described as a key regulatory element in response to stress^30,31^.

Cancer is another life-threatening disease that causes elevated lactate levels. It is known that cancer cell metabolism is different from normal cells, and demonstrate an abnormal conversion of glucose to lactate, even in the presence of oxygen (aerobic glycolysis)^9–12^. A sudden change in the balance between glycolysis and oxidative metabolism can also take place as a result of important metabolic changes^32^.

Given this overview, it is not surprising that there is overwhelming evidence that underlines the relationship between increased lactate levels and increased morbidity and mortality in critically ill patients. This further highlights the importance of monitoring lactate in the early resuscitation of critically ill patients^1,2,26^. These subjects have been more extensively explored in the litrature^26,33–36^.

### Spectroscopic data and the small $n$, large $p$ problem

In spectroscopic applications, commonly one is interested in analysing the optical properties of a molecule over a wide wavelength interval. Such intervals can include hundreds or thousands of wavelengths (variables, $p$). In quantitative analysis, the target may be finding a mathematical model that maps such spectra to the concentration of an analyte. However, the time and cost associated with sample preparations and obtaining the spectra often limits the number of spectra (observations, $n$) to only tens. This poses a challenge for model training and is commonly known as the small $n,$ large $p$ problem.

Partial Least Squares (PLS) and Principle Component Regression (PCR) are commonly used in the analysis of spectroscopic data due to two reasons. Firstly, often many of the wavelengths in the spectra contain collinear, redundant or uninformative information and both PLS and PCR can adequately eliminate the negative effects of such variables. They reduce the dimensionality of the $p$-dimensional data -the spectra- by projecting them onto a $c$-dimensional space, where $c<p$. Generally, the larger the number of collinear or redundant variables, the smaller can $c$ become. The resulting variables are known as latent variables. Secondly, based on the Beer-Lambert law, there is a linear relationship between the optical absorbance of an absorbing species and its molar concentration, when the concentration is low. Both PLS and PCR are linear—or more accurately bilinear—regression methods.

The predictive ability of PLS and PCR are often shown to be very similar in practical applications. However, PLS usually achieves the same level of accuracy with less latent variables. This is due to the fact that PLS maximises the covariance between independent variables, $X$, and dependent variables, $Y$, while Principle Component Analysis (PCA) finds the axes of maximal variance in the independent variable space (eigenvectors), $X$ ^37–39^. While PLS is used throughout this study, the methods discussed here are generalisable to PCR. Moreover, apart from the first two investigated methods, i.e. the methods suggested by Frenich et al.^40^ and Centner et al.^22^ which rely on the coefficients of independent variables in the linear model, the rest of the methods can be generalised to nonlinear regression methods such as random forests, gradient boosted trees, support vector regression and neural networks.

PLS can be described using Eqs. (1) and (2).

1 $$X=T{P}^{T}+E$$

2 $$Y=U{Q}^{T}+F$$

where $X$ denotes the ($n\times p$) matrix of independent variables e. g. the absorption profiles for $n$ samples, each including $p$ wavelengths, $Y$ denotes the ($n\times m$) matrix of dependent variables, e.g. the concentrations of $m$ absorbing species for $n$ spectra, $T$ and $U$ are ($n\times c$) matrices of latent variables, also known as $X$- and $Y$-score matrices, or PLS components, $P$ and $Q$ are known as $X$ and $Y$-loading matrices and are ($p\times c$) and ($m\times c$) respectively, $E$ is the ($n\times p$) matrix of residuals for $X$ and $F$ is the ($n\times m$) matrix of residuals for $Y$. PLS maximises the covariance between the latent variables of $X$ and $Y$ as represented by the $T$ and $U$ matrices, $cov(t,u)$. Assuming a linear relationship between matrices $T$ and $U$ would lead to,

3 $$Y=XB+G$$

where $B$ is the ($p\times m$) matrix of regression coefficients and $G$ is the $(n\times m)$ matrix of regression residuals^41^. The choice of the number of latent variables can determine overfitting or under-fitting. While different criteria for the selection of the number of latent variables have been proposed in the literature, a common approach is to plot the Predicted Residual Error Sum of Squares (PRESS) against the number of latent variables and choosing the point where the PRESS plateaus^39^.

## Dataset

This section describes the spectra collection procedure and has previously been comprehensively described^42^. Na-lactate and isotonic PBS were acquired in dry form from Thermo Fisher Scientific (Massachusetts, USA). A stock solution of 600 mmol/L Na-lactate was prepared by dissolving 67.236 g of Na-lactate powder in 1L of deionized water. The lactate stock solution was then diluted with 1X aqueous PBS to make 47 solutions with molar concentrations of lactate ranging between 0 to 18 mmol/L. The lactate increments are 0.25 mmol/L within the range of 0–5 mmol/L (21 samples), and 0.5 mmol/L within the range of 5–18 mmol/L (26 samples).

All solutions were maintained at room temperature 24 °C ($\pm 0.5$), and their potential of Hydrogen (pH) values was tested to be 6.5 ($\pm 0.2$). Measurement of pH was carried out using Orion Star A211 Advanced pH Benchtop Meter Kit (Thermo Fisher Scientific, Massachusetts, USA). The concentrations of lactate were verified using an LM5 lactate analyzer (Analox Instruments Limited, Stourbridge, England, UK).

FTIR spectra of samples, within 450–4000 $c{m}^{-1}$ (2500–22,222 nm), were obtained using Spectrum Two FTIR Spectrometer (Perkin Elmer, Massachusetts, USA) with horizontal Attenuated Total Reflectance (ATR) accessory-ZnSe crystal (Pike Technologies, Madison, WI, USA) and the following settings, resolution of $4 c{m}^{-1}$, an interval of $1 c{m}^{-1}$, accumulation of 100 scans, and internal Jacquinot-stop of 8.94 $mm$. The lactate solutions were run at random to prevent any potential temporal bias.

**Figure 1:**
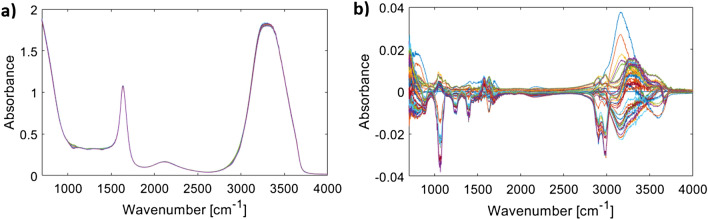
The spectra of the dataset containing 47 ATR-FTIR spectra of lactate in PBS solutions. (**a**) Shows the 47 spectra of lactate in PBS, overlaid due to the dominant effect of water absorption peaks in the solutions. (**b**) Highlights the changes in absorption of lactate by subtraction of base lactate (0 mmol/l).

All pre-processing operations and implementation of algorithms were carried out in MATLAB R2018a. Figure 1, depicts the dataset consisting of 47 ATR-FTIR spectra of lactate in PBS solutions. The concentrations of lactate vary between 0–18 mmol/L. Figure 1a shows the raw absorbance spectra, Fig. 1b shows the spectra with the PBS spectrum subtracted.

## Wavelength selection

Although PLS and PCR address some of the issues that rise from the large $p$, small $n$ nature of spectroscopic data, it has been shown that the inclusion of uninformative wavelengths in the training process negatively affects the accuracy of predictions as well as model interpretability^19–22,43^. Moreover, from a more practical point of view, the identification of the specific wavelengths, or regions of the optical spectrum, that contain information about chemical species, significantly reduces the time and cost associated with monitoring them and facilitates the development of portable, high-speed sensors.

The straightforward application of statistical variable selection techniques, such as forward selection and backward selection, to small $n$, large $p$ problems can lead to extreme overfitting. This is due to the excessive model selection/rejection steps, which could exponentially increase with the number of variables for some methods. This excessive selection/rejection can undermine statistical measure of significance, cause type II errors, and lead to overfitting^44,45^.

What follows provides a brief overview of some wavelength selection methods. In particular, wavelength selection methods may be categorised into three main groups (1) simple strategies for selection/elimination of variables based on the variables’ weights (coefficients) in predictive models (2) interval selection methods and (3) heuristic global search methods. These methods are briefly described below. Subsequently, some of the well-known methods from each group are applied to the lactate dataset and the results are compared.

Throughout this article, in order to ensure that validation sets are sufficiently representative of the dataset, they are chosen in the following way. To select ${n}_{v}$ data points for validation, the dataset is first sorted based on the concentration of lactate and is then split into ${n}_{v}$ blocks, such that all blocks contain an equal number of spectra or one less when the number of spectra is not divisible to ${n}_{v}$. Subsequently, a data point is randomly selected from each block to produce the required ${n}_{v}$ validation data points.

The validity of models is assessed using cross-validation and an independent test set. Due to the need for manual intervention in some cases as well as the computationally demanding nature of some of the investigated wavelength selection methods, it is impractical to perform wavelength selection within each cross-validation loop. Therefore, cross-validation is performed on the models with selected wavelengths. This is expected to bias the cross-validation results and underestimate RMSE due to data leakage. To mitigate this issue, Monte Carlo cross-validation (MCCV) is used. In MCCV, a dataset containing $n$ samples is randomly split into a validation set of size ${n}_{v}$ and a calibration set of size $n-{n}_{v}$. The model is then calibrated on the $n-{n}_{v}$ observations and predication errors are calculated for the ${n}_{v}$ held-out observations. This process is repeated for a predetermined number of times ($N$) to estimate the prediction error. RMSECV_MC_ is defined as,

4 $$RMSEC{V}_{MC}=\sqrt{\frac{1}{N\times {n}_{v}}\sum_{i=1}^{N}\sum_{j=1}^{{n}_{v}}{\left({y}_{ij}-{\widehat{y}}_{ij}\right)}^{2}},$$

where ${\widehat{y}}_{ij}$ is the predicted value of the jth data point in the validation set and in the ith iteration of MCCV and ${y}_{ij}$ is the associated reference value. Compared to the two widely used leave-one-out and leave-${n}_{v}$-out (${n}_{v}>1$) cross-validation routines, MCCV (similar to leave-${n}_{v}$-out) places more emphasis on the validation process by including more than one observation in the validation set. In leave-${n}_{v}$-out, however, all of the possible ways in which the data can be split into calibration and validation sets need to be examined ($i.e. {C}_{{n}_{v}}^{n}=\left(\genfrac{}{}{0pt}{}{n}{{n}_{v}}\right)$ events), while in MCCV only a random subsample of such events is examined. Therefore, MCCV is more computationally tractable than leave-${n}_{v}$-out and less susceptible to overfitting than leave-one-out^46^. Finally, the cross-validation results are complemented with the prediction results, RMSEP and coefficient of determination %R^2^_Test_, pertaining to a representative independent test set of eight, held out from all pre-processing and wavelength selection steps, and only used for the validation of the models with selected features. RMSEP is given by,

5 $$RMSEP=\sqrt{\frac{1}{{n}_{t}}\sum_{i=1}^{{n}_{t}}{\left({y}_{i}-{\widehat{y}}_{i}\right)}^{2}}, $$

where ${n}_{t}$ denotes the number of observations in the independent test set. %R^2^_Test_ is defined as,

6 $${R}_{Test}^{2}=\left(1-\frac{\sum_{i=1}^{{n}_{t}}{\left({y}_{i}-{\widehat{y}}_{i}\right)}^{2}}{\sum_{i=1}^{{n}_{t}}{\left({y}_{i}-\stackrel{-}{y}\right)}^{2}}\right)\times 100 ,$$

where $\stackrel{-}{y}$ is the mean of the dependent variable in the test set.

### Coefficient-based variable importance

Garrido Frenich et al. proposed a simple method based on PLS regression coefficients ($b$)^40^. In this method, similar to the interpretation of parameters in standard linear regression, the PLS coefficients—$B$ in Eq. (3)—are considered as a measure of variable (wavelength) significance, although in small $n$, large $p$ problems there is little statistical justification to do so, and type I errors are to be expected. Moreover, a large coefficient can also represent a variable with small absolute value and large variance. In order to mitigate the error that could be caused by such variables, each variable is scaled with the inverse of its standard deviation. The variables with high regression coefficients, ${b}_{i}^{^{\prime}}$ , for the standardised matrix, ${X}_{standardised}$, are considered to be important variables^40^, where ${b}_{i}^{^{\prime}}$ is the element of the coefficient vector $B{^{\prime}}$ that corresponds to the variable $i$.

**Figure 2:**
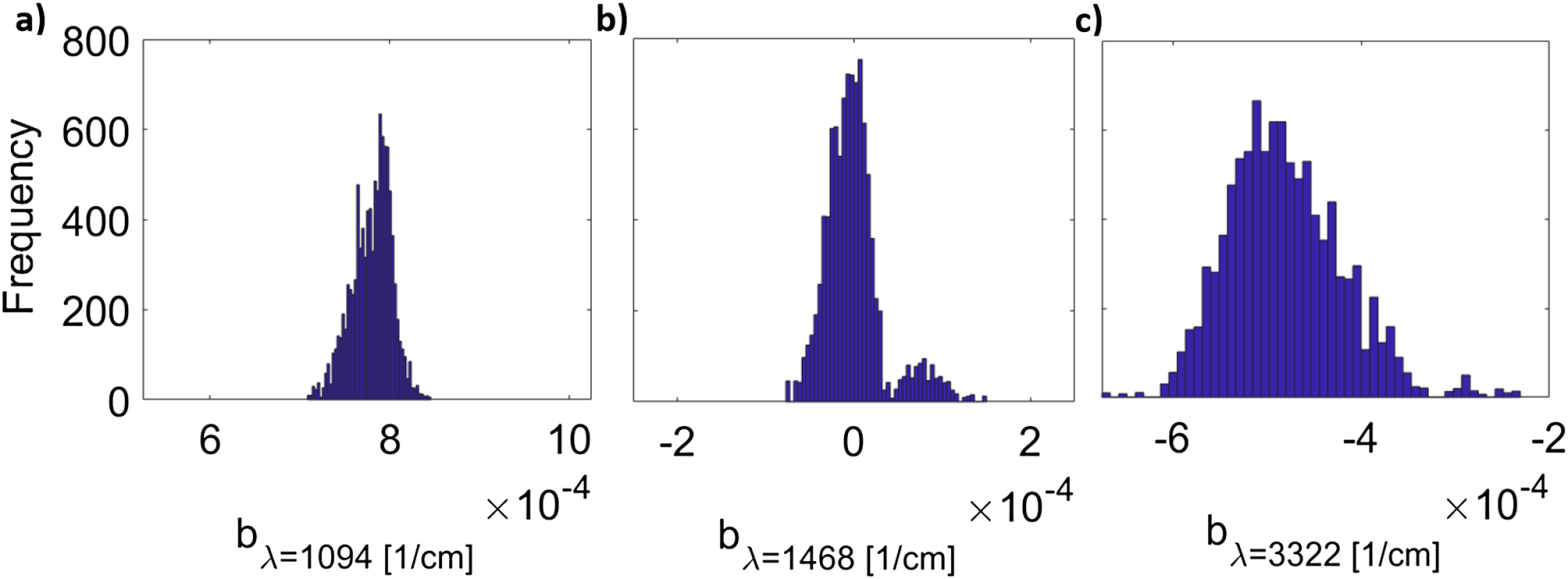
Histograms of the PLS coefficients for wavelengths (**a**) 1094, (b) 1468, c) 3322 [${\mathrm{cm}}^{-1}$] obtained using Monte Carlo cross-validation with 10,000 iterations.

A disadvantage of the method described above is that it does not consider the uncertainty of $b{^{\prime}}$ coefficients. In particular, these values can exhibit drastic changes in successive training with resampled data. To demonstrate this, the histograms of three coefficients obtained with MCCV for the lactate dataset are depicted in Fig. 2.

Centner et al. proposed a method, Elimination of Uninformative Variables (EUV) that similarly uses the regression coefficient to assess the importance of variables^22^. The EUV captures the uncertainty of ${b}_{i}^{^{\prime}}$ coefficients by considering a reliability measure, that is equivalent to the t-static for the null hypothesis of ${H}_{0}$: ${\mu }_{{b}_{i}}=0$,

7 $${c}_{i}=\frac{\stackrel{-}{{b}_{i}}}{\sigma \left({b}_{i}\right)} ,$$

where ${c}_{i}$ is the reliability of the variable $i$, $\stackrel{-}{{b}_{i}}$ and $\sigma ({b}_{i})$ are the mean and standard deviation of the PLS regression coefficient for the ${i}$th variable (wavelength). These estimates are obtained using a resampling method, for instance, leave-one-out. The reliability values below a certain threshold may be deemed uninformative and, therefore, unselected. To find a good threshold, the spectra are augmented with random values and the cut-off reliability is selected as $\mathrm{max}\left(abs({c}_{artif})\right)$. The application of this method to the lactate dataset leads to the elimination of almost 70% of variables.

### Interval selection techniques

The optical spectra have locality features. In other words, the information about the concentration of chemical species is focused on certain regions of the optical spectra. The incorporation of this assumption into the search strategies yields a different class of wavelength selection techniques. A simple way to achieve this is to split the spectra into equidistant intervals and treat each interval as a variable. As a result, the dimensionality of the space of variables can be significantly reduced, from thousands of variables to tens of intervals. This has the additional benefit that many classical techniques such as Forward Selection (FS) and Backward Elimination (BE) can be applied to these intervals. This is the key insight behind interval PLS (iPLS) proposed by Nørgaard et al. ^47^, and its variations such as forward iPLS (combination of FS and iPLS), backward iPLS (BE and iPLS), synergy iPLS (combinatorial interval selection), and GA-iPLS (selection of interval combinations using a genetic algorithm). Nørgaard et al. also suggested micro-optimisations, such as one-sided or double-sided expansion and truncation of the “best interval”. The number of intervals is a key parameter that could have major implications on the outcome. In this study, two values for the number of intervals were used, 20 and 40.

While iPLS can provide a good representation of the informative regions of the optical spectrum, the two requirements of equidistant intervals and the predetermined number of intervals are restrictive. As a result, there is very little chance that the optimal interval can be found using this approach. Moving Window PLS (MWPLS) relaxes these requirements by selecting an interval with a predetermined length at one end of the spectrum and then moving the centre of the window one wavelength at a time^48^. As an alternative to RMSECV and RMSEV, Jiang et al. suggested a criterion based on the uncertainty of the local models. This uncertainty is reflected in the number of latent structures (the number of PLS components) and the Sum of Squared Residuals (SSR) of training. Multiple models with different complexities (PLS components) are developed for every interval. The goodness of an interval is then judged by how the addition of PLS components improves the SSR. If the SSR is relatively high or if it continues to significantly improve with the addition of PLS components, this suggests that these components are used to model inherent uncertainties, and therefore, the interval is not informative. In summary, according to this criterion, a good interval is an interval that obtains low SSRs with only a few components and the addition of extra components does not significantly improve the results.

### Heuristic global optimisation search methods

A different approach is the use of heuristic global optimisation methods to find a suboptimal combination of variables. Here the problem can be formulated as an optimisation problem where the objective is to minimise a cost function, such as RMSE of Calibration (RMSEC), or RMSECV, by choosing the right combination of variables. Genetic algorithms, a class of biologically-inspired, evolutionary algorithms have found many applications engineering, biomedicine, chemometrics, genomics, and spectroscopy due to their ability in solving complex, nonlinear optimisation problems. Many variations and different implementation of this method can be found in the literature^49–55^.

Genetic algorithms are inspired by Darwinian selection, i.e. the survival of “good” genes in offspring. The algorithm begins with a random population of chromosomes or candidate solutions. In a variable selection problem, each chromosome can be coded as a bit-string, where a one represents selection and a zero represents deselection. Each chromosome is then assigned a fitness value or an unfitness value in a minimisation problem. Here the unfitness value can be RMSEV, RMSEC, RMSECV. Candidate solutions (chromosomes) that produce better results will have a higher likelihood of passing on their genes to the next generation (selection), recombining their -likely good- genes with other fit genomes (crossover), and finally passing on their genes to the next generation of genomes with some random mutation. The last operation is necessary to ensure that the optimisation landscape will be explored and reduce the likelihood of getting stuck in local minima. This process will continue for a predetermined number of generations or until a convergence criterion is met.

#### The proposed GA method

In large $p$ small $n$ problems, genetic algorithms are likely to lead to over-fitting. A rule of thumb to avoid this is to keep the ratio of the number of variables, $p$, and the number samples, $n$, small. It has been suggested that depending on the noise levels the ratios ($p/n$) of greater than five are very likely to lead to overfitting^55^. In the lactate dataset, this ratio is greater than 80, i.e. 3151 wavelengths and 39 samples in the training set. However, there are some measures that can mitigate this issue, such as using intervals rather than variables (similar to iPLS) or using a dependant validation set to stop the operations when the RMSEV starts to increase (early stopping). As mentioned in Sect. 4.2, using intervals imposes extremely limiting constraints which leaves very little chance of obtaining a minimal and optimal selection of wavelengths. Therefore, in the proposed method we use the bit-string representation of wavelengths. The unfitness function is defined as MSEC, however, in order to reduce the likelihood of over-fitting three strategies are incorporated in the GA method;

- Firstly, in each run of GA, the training set is randomly split into a validation set and a training set. The RMSEV for the randomly selected validation set is only used once to evaluate the goodness of the final solution for that GA run. If the final solution obtained in a GA run produces a RMSEV above a threshold, it will be discarded. Here the threshold is defined as $1.5\times RMSE{V}_{full model}$.
- Secondly, the evaluation of the fitness function for each chromosome is done using MCCV with 100 resampling iterations. In particular, the training set is randomly split into a training set and a validation set and the MSEC and MSEV are calculated. This resampling process is repeated 100 times and the resulting MSECs and MSEVs are averaged to produce a more robust estimate of MSEC and MSEV , denoted by  $\overline{{MSEC}}$ and $\overline{{MSEV}}$ for a given chromosome. This layered design minimises the likelihood of overfitting since it ensures that the solution has produced good results across thousands of resampled datasets (a) in every fitness evaluation step and (b) along different generations. Moreover, it produces a number of important statistics that can be incorporated in the objective function, for example, the mean and standard deviations of the MSEC and/or MSEV. In the current study, only the average estimate of MSEC is used in the objective function, but a comprehensive analysis of different objective functions will be carried out in the future.
- Finally, the genetic algorithm is run 100 times, and the importance of variables are calculated as the average number of times that a variable is selected.

**Figure 3:**
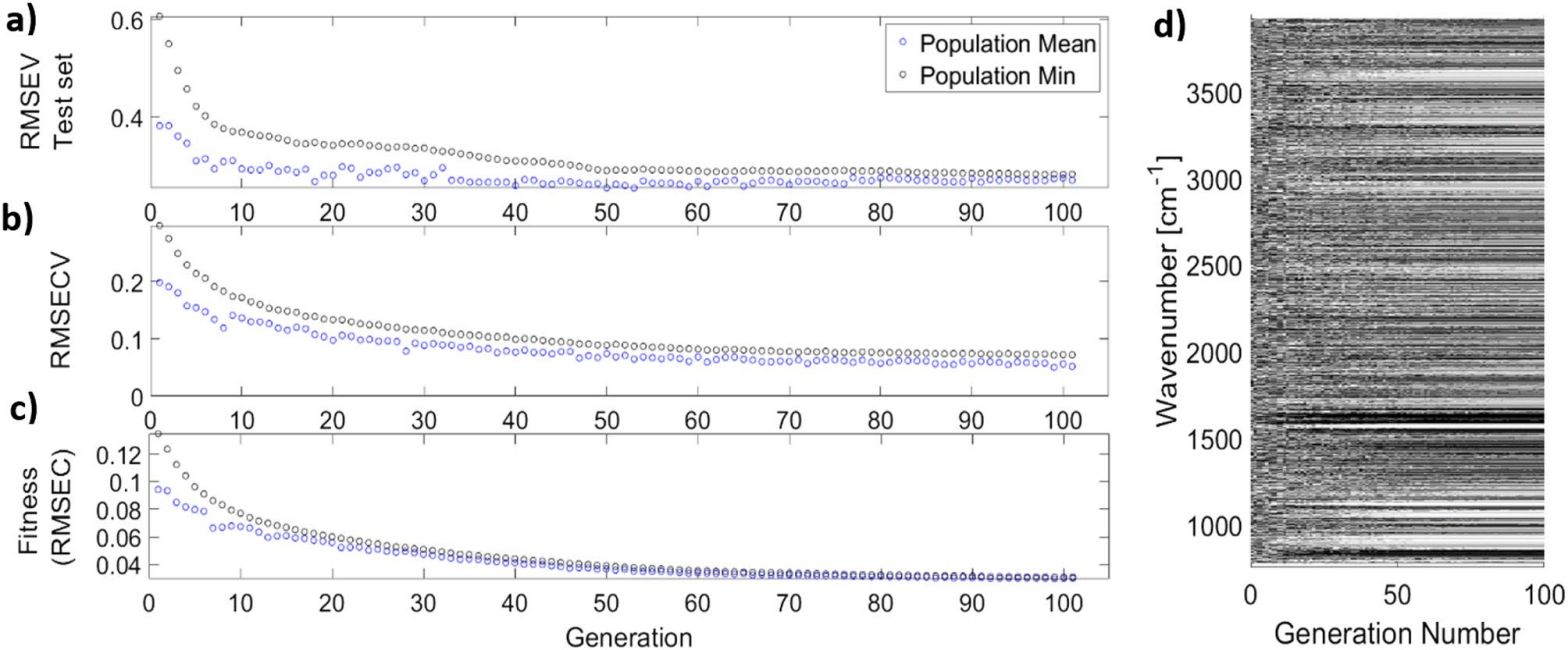
Application of the proposed GA-based wavelength selection method to the lactate dataset. The figures pertain to one run of the GA. (**a**) RMSEV for the GA run validation set (**b**) RMSECV, (**c**) the fitness value (RMSEC), (**d**) The convergence of the selected wavelengths from an initial random population to a solution after 100 generation, the black marks represent selection.

Figure 3 depicts the results for one run of the proposed GA-based wavelength selection on the lactate dataset. Figure 3c demonstrates a smooth convergence to a minimum, validating the choice of $\overline{{RMSEC}}$ as a measure of chromosome unfitness. Figure 3a,b show $RMSEV$ and $\overline{{RMSECV}}_{MCCV}$ respectively, both suggesting that the proposed method successfully avoids overfitting. Finally, Fig. 3d shows the selection of wavelengths (denoted with black pixels), in the fittest chromosome of the generation. It can be seen that the initial randomly selected variables in the first generation gradually converge to a solution.

**Figure 4:**
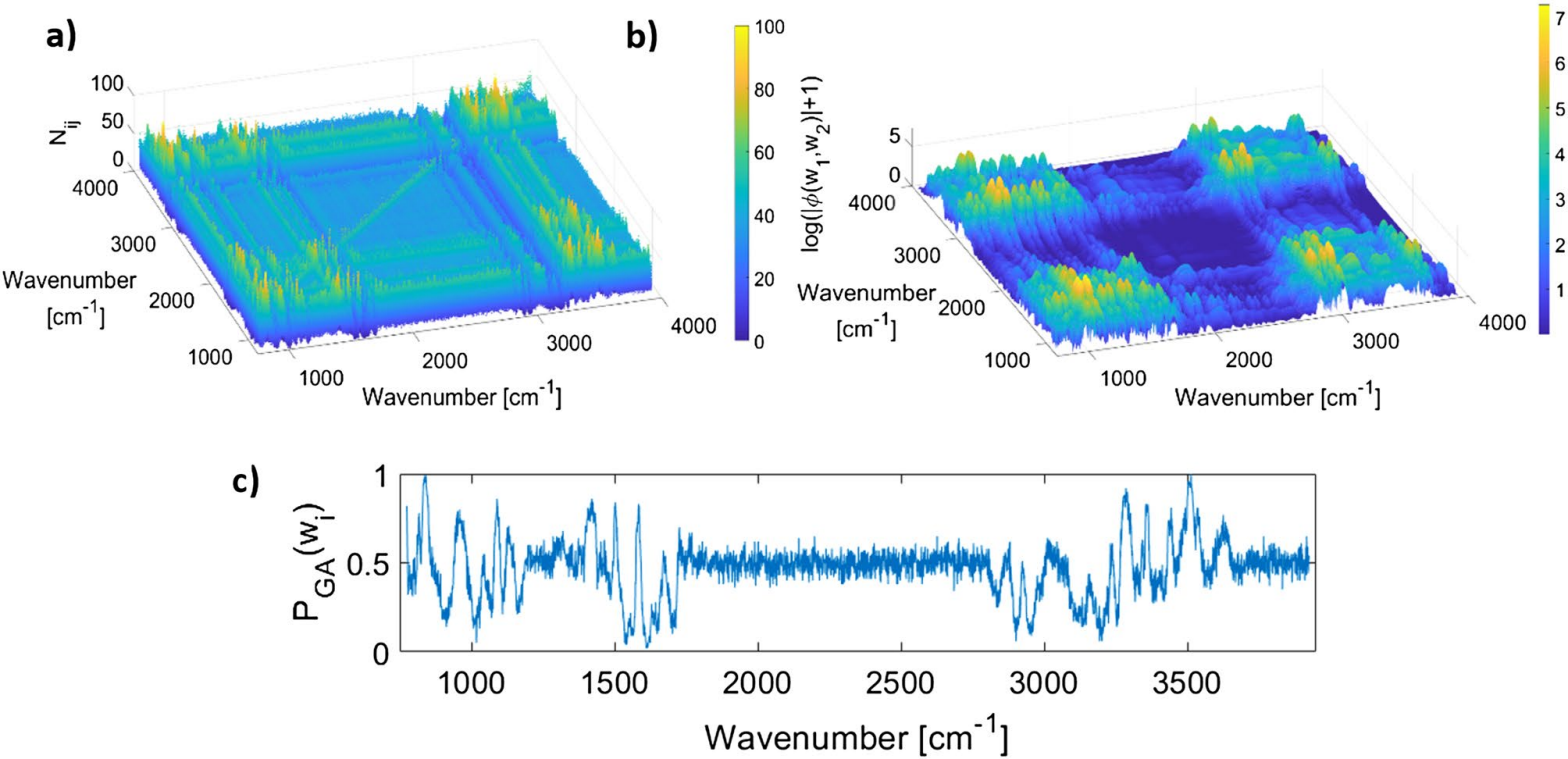
(**a**, **b**) The comparison of the H matrix and the 2D correlation synchronous plot for the lactate dataset. (**c**) The average number of times that each wavelength was selected.

In order to ensure that the solutions in different runs are stable, they are arranged in rows of a matrix, ${G}_{n\times p}$. The matrix $H={G}^{t}\times G$, is a $(p\times p)$ matrix, where ${G}^{t}$ is the transpose of $G$. Each element of this matrix, ${h}_{ij}$, shows the number of times that wavelength $j$ was selected when wavelength $i$ was selected. Some similarities between matrix $H$ and 2D correlation synchronous plot are to be expected, however, matrix $H$ highlights 1. wavelengths that most contribute to the prediction of lactate given the underlying model (here PLS) 2. Shows how consistent the pattern of selection deselection in different runs of the GA are. The matrix H and the 2D correlation synchronous plot are depicted in Fig. 4a,b. The probability of selection in different runs of GA for wavelength ${w}_{i}$ is denoted with ${P}_{GA}\left({w}_{i}\right)$ and is defined as the number of times that ${w}_{i}$ was selected divided by the number of GA runs. This is depicted in Fig. 4c.

Interestingly, it can be seen that while unlike iPLS and MWPLS, the locality of features is not hardcoded in the method, local features have emerged, i.e. adjacent wavelengths have relatively similar ${P}_{GA}({w}_{i})$ values. Moreover, the results are much more granular and easier to interpret compared to both the aforementioned methods. Apart from the major peaks in the finger print region (i.e. $500-1500 c{m}^{-1}$ ) the region between $3260-3650$ $c{m}^{-1}$ contains a few significant intervals, particularly the interval $3490-3520$ $c{m}^{-1}$ which has previously been linked to α-hydroxy-esters methyl lactate^56^.

## Results

**Figure 5:**
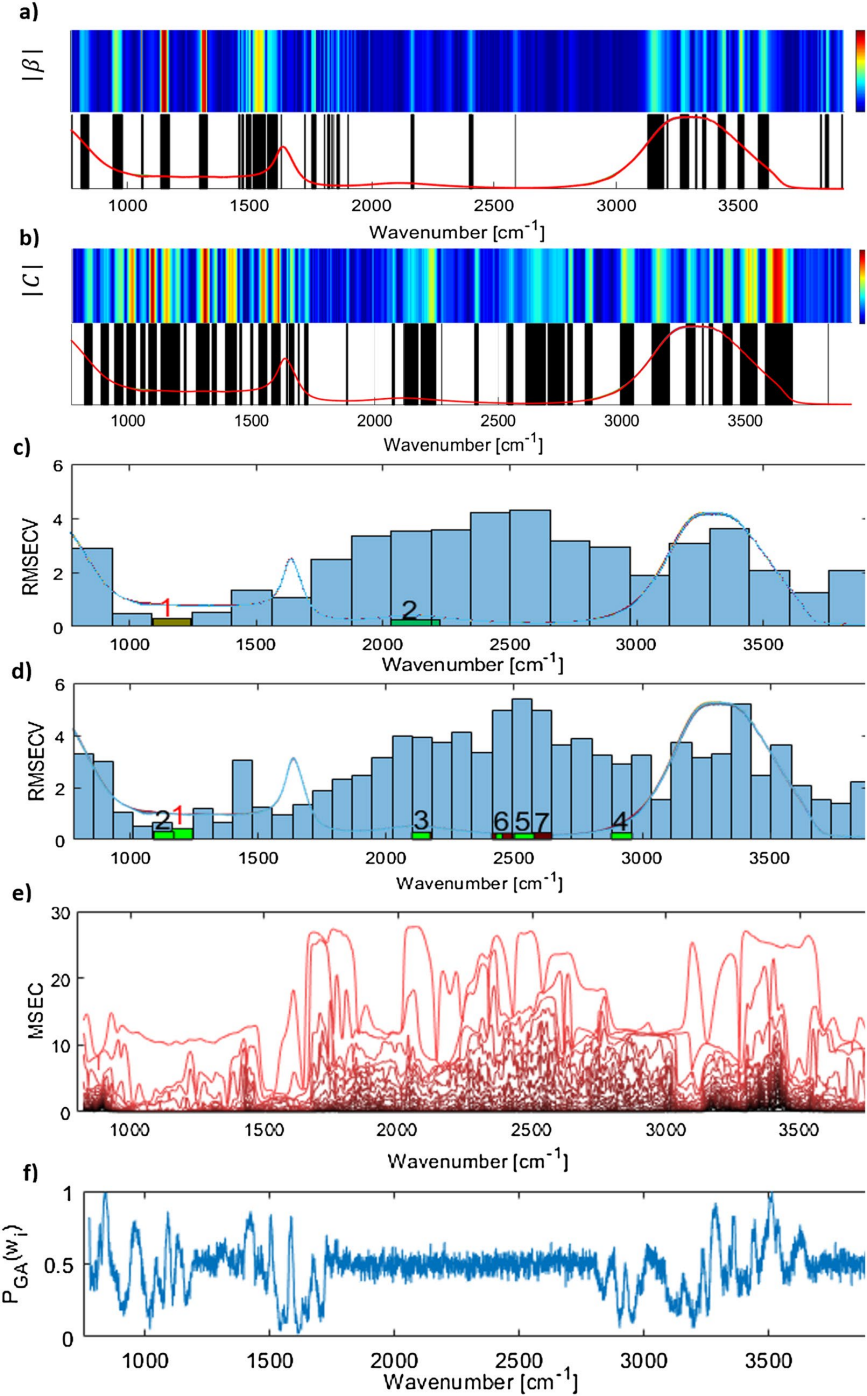
(**a**, **b**) The method proposed by Garrido Frenich et al.^40^ and EUV respectively. The upper part of the plot depicts the ${\mathrm{b}}_{\mathrm{i}}\mathrm{^{\prime}}$ (in Fig. 5a) and ${\mathrm{c}}_{\mathrm{i}}$ (in Fig. 5b) values for all wavelengths. The lower part depicts the selected wavelengths. (**c**, **d**) The blue bars depict RCMECV for each interval for IPLS20 and IPLS40 respectively. The green bars and the numbers above them demonstrate RMSECV in FS_IPLS and the order in which the intervals were selected. The green bars can be narrower, thicker, or shift to the sides depending on the subsequent micro-optimisations. (**e**) MWPLS: MSEC for local models with a different number of components. The darker lines depict models with more components. (**f**) ${\mathrm{P}}_{\mathrm{GA}}({\mathrm{w}}_{\mathrm{i}})$ for all wavelengths in the proposed GA-based wavelength selection method.

In this section, the eight reduced models, and the complete model are compared. The model based on the interval selected by iPLS with 20 intervals and 40 intervals are denoted by iPLS20 and iPLS40 respectively. The two FS-iPLS models are similarly denoted by FS-iPLS20 and FS-iPLS40. The method presented in Garrido Frenich et al., is denoted with $\beta$. Prior to the analysis the optimal number of components for all 12 models are selected as the elbow point in the Predicted Residuals Error Sum of Squares (PRESS) plot. The model based on MWPLS uses the interval of 1050–1430 $c{m}^{-1}$ which is chosen based on the criterion set out Jiang et al.^48^. For the proposed GA model, all wavelengths with ${P}_{GA}({w}_{i})$ smaller than 0.6 have been eliminated to produce a reasonable reduction in the number of wavelengths. A more rigorous approach to select this threshold could be the use of RMSECV and RMSEV, but in the present study, this has not been considered for simplicity. Figure 5 demonstrates the variables selected by each method.

The comparison of the nine models is summarised in Table 1. RMSECV is obtained using MCCV and on models with predetermined wavelengths. 10,000 resampling iterations are used and in each iteration 5 samples are held out for validation and the remaining 42 samples are used for training.

**Table 1:** The comparison of the parsimonious models derived using different wavelength selection methods in the estimation of lactate concentration. RMSECV is obtained for the models with selected features using a Monte Carlo cross-validation with 10,000 resampling iterations. ${\mathrm{RMSEP}}_{\mathrm{Test}}$ is obtained for an independent test set of 8 randomly selected spectra. ${\mathrm{R}}_{\mathrm{Test}}^{2}\mathrm{ \%}$ is the coefficient of determination for the test set.

Method	# vars	#LVs	$RMSECV [mmol/L]$	$RMSE{P}_{Test}$ $[mmol/L]$	${R}_{Test}^{2} \%$
$Full-PLS$	3151	6	0.4486	0.2935	99.7570
$\beta$	630	7	0.4562	0.3825	99.5873
$EUV$	1071	6	0.3634	0.3370	99.6797
$iPLS20$	151	7	0.2933	0.2665	99.7997
$iPLS40$	84	7	0.4419	0.5305	99.2063
$FS\_iPLS20$	345	8	0.2422	0.2195	99.8641
$FS\_iPLS40$	424	10	0.2353	0.2609	99.8079
$MWPLS$	379	7	0.3576	0.3791	99.5945
$G{A}_{P>0.6}$	415	7	0.3868	0.3654	99.6234

Table 1 demonstrates the potential of variable selection methods for obtaining more accurate predictions while relying on a much smaller number of variables. The method proposed by Garrido Frenich et al., denoted by $\beta$, obtains a comparable performance with the complete model while significantly reducing the number of variables. The EUV helps eliminate two-third of uninformative variables while reducing the RMSECV compared to the complete model and delivering a comparable RMSEP_Test_.

The iPLS20 model exceeds the predictive ability of the complete model while only using the interval of 1090–1240 cm^−1^. This interval is associated with the stretching modes of C–C, C–O, C–O–C, and C–OH functional groups. The iPLS40 selects the interval 1160–1243 cm^−1^ and provides a comparable RMSECV with the complete model, although the RMSEP is notably higher than all other models. The two FS-iPLS models outperform all other models, although this comes at the cost of higher model complexity. MWPLS does not identify clearly defined intervals, but rather helps infer more informative intervals. The model inferred from the application of MWPLS, 1050–1430 cm^−1^, covers a section of the fingerprint region that in addition to the previously mentioned functional groups includes O–H bending.

Interval selection methods such as IPLS and MWPLS are highly constrained by the locality assumption. These methods iteratively evaluate local models and completely ignore the potentially significant effects of combining non-neighbouring wavelengths (a) in the assessment of wavelength importance, and (b) in the determination of the optimal combination of wavelengths for quantitative analysis. Modifications such as FS-IPLS to some extent address this limitation, however, as it can be seen from Fig. 5c,d firstly they do not provide a comprehensive interpretation of all relevant wavelengths since they only search through a small subset of all possible combinations and ignore $k$-combinations, $k>1$. Secondly, the selection criterion is highly dependent on the choice of the number of intervals. The method proposed by Garrido Frenich et al., $\beta$, and EUV do not suffer from the restrictive assumptions of interval selection methods. They provide a good understanding of all relevant wavelengths -although with potentially many false positives-, but they fully ignore the combinatorial importance of variables. Also, the wavelengths selected based on these methods can be many and spread across the spectrum, therefore, they might be of little help for developing optical sensors with minimal wavelengths.

The proposed GA based method provides a detailed and unconstrained measure of combinatorial importance of variables (Fig. 5f). It can also be seen from Table 1 that a good trade-off between the number of variables and the accuracy of predictions can be achieved by selecting variables that were in the final solution in more than 60% of GA runs. However, the simple strategy of only selecting wavelengths that appear in most of the GA run solutions is outperformed by PLS20, iPLS20-FS, and iPLS40-FS. It is possible a combination of these variables and less frequently selected variables may be necessary to deliver predictions with higher accuracies.

## Conclusion

A comprehensive investigation of the application of wavelength selection methods was carried out on a lactate and PBS dataset and it was shown that significant reductions in the number of required wavelengths is not only possible but could also be the key for achieving higher accuracies. Additionally, a new genetic-algorithm based variable selection method was proposed and it was shown that the method can provide an unconstrained and granular interpretation of wavelength importance. The proposed GA method successfully avoids overfitting, provides stable solutions, and provides a valuable interpretation of the combinatorial importance of wavelengths. However, the simple strategy used here for the extraction of variables from the GA method does not provide the highest predictive ability amongst the investigated methods. To address this, our future effort will focus on the incorporation of model parsimony in the objective function.

Moreover, in our future work, more complex matrices, namely serum, and blood will be investigated. In these solutions, more scatterers and absorbing species with similar molecular structures to lactate are present. The former can lead to a lower signal-to-noise ratio and the latter can result in overlapping peaks between these absorbers and lactate. Additionally, the interaction of lactate with other chemical entities in the blood can complicate the analysis. Nevertheless, as mentioned in the introduction section, previous studies that have used broad regions of the optical spectrum for estimation of lactate in blood, have reported promising results. The authors expect that the comparison of wavelengths pertinent to the prediction of lactate in PBS and in blood, will help conclude which of those are more suitable for practical applications. Finally, since the NIR region of the optical spectrum is more appropriate for transcutaneous applications, similar studies will be carried out with a focus on the NIR region.
